# Glycogen-rich clear cell carcinoma of the breast

**DOI:** 10.1186/1477-7819-6-44

**Published:** 2008-04-29

**Authors:** Christos Markopoulos, Dimitris Mantas, T Philipidis, Efstatios Kouskos, Zoi Antonopoulou, ML Hatzinikolaou, Helen Gogas

**Affiliations:** 1 Breast Unit, 2^nd ^Propedeutic Department of Surgery, Athens University Medical School, Greece

## Abstract

**Background:**

Glycogen-rich carcinoma of the breast is a rare histological subtype of breast cancer, usually reported to have poor prognosis.

**Case presentation:**

We present the case of a 59-year-old woman who underwent a mastectomy for a 3.5 cm clinically palpable left breast carcinoma, originally diagnosed as fibroadenoma on a screening mammogram four years before presentation. Diagnosis of clear cell carcinoma was based on certain histological characteristics of the tumour and immunohistochemical analysis (PAS staining, keratins AE1/AE3, EMA, cytokeratin 7, cytokeratin 20, melanosomes, vimentin, Chromogranin, Synaptophysin, S-100, SMA). No lymph node metastasis was found and as the tumour was ER positive and PgR negative, patient was treated only with an aromatase inhibitor upfront and remains free of disease 48 months now since operation.

**Conclusion:**

Glycogen-rich clear cell carcinoma of the breast is a rare tumor, its clinical behavior reported to be rather aggressive so far, might varies depending on special characteristics such as low grade and strongly positive ER expression

## Background

Glycogen-rich clear cell carcinoma is a rare neoplasm of the breast, with an incidence of between 1.4% and 3% of all breast cancers [1,2]. The tumour has distinct morphology, different from that of common breast cancers. It shares common characteristics with clear cell carcinomas of the lung, endometrium, cervix, ovary, kidneys and salivary glands [3]. Glycogen-rich clear cell carcinomas are members of a heterogeneous group of neoplasms, including signet-ring, secretory and lipid-rich carcinomas of the breast [4]. In general, clear cell breast carcinoma tends to follow an aggressive clinical course [5]. However, we report the case of a 59-year-old woman with a slow growing tumour of her left breast, originally considered as fibroadenoma, but which proved to be a 3.5 cm glycogen-rich clear cell carcinoma without lymph node involvement, four years later.

## Case presentation

**Figure 1:**
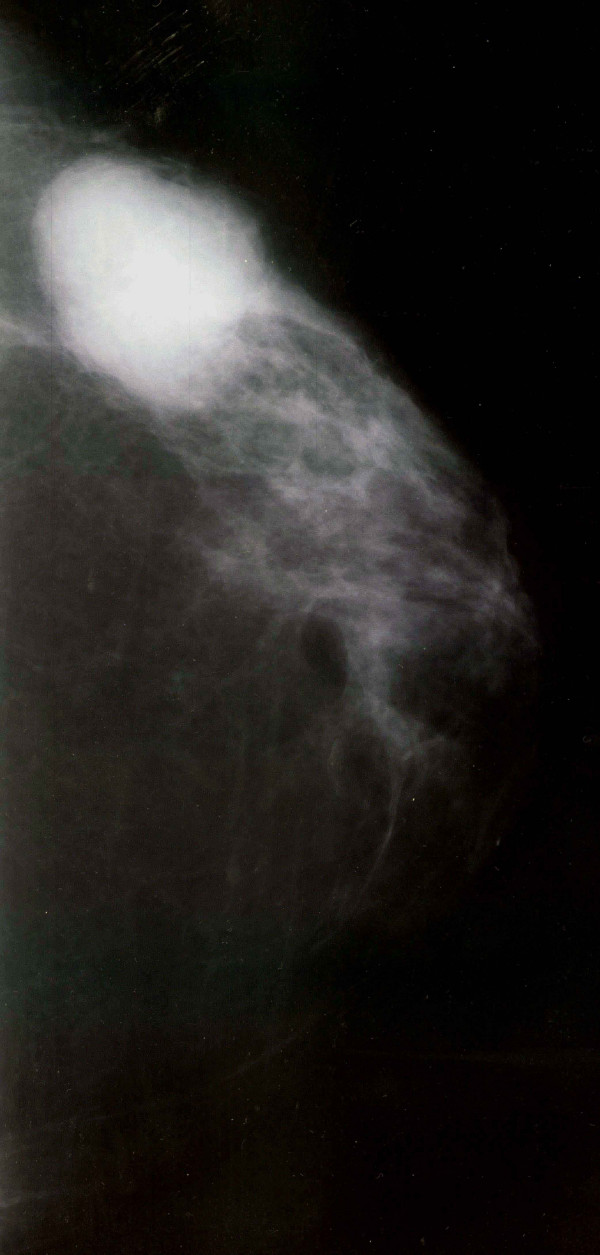
Left breast mammogram showing a lobulated, circumscribed mass with no evidence of malignancy, suggestive of a fibroadenoma.

A 59-year-old Caucasian woman presented with a breast mass in the upper outer quadrant of her left breast. She noticed the lump on self-examination a few months before presenting to our out-patient clinic. The lesion was mobile, with no evidence of dermal invasion and axillary lymph nodes were not palpable. A 3.5 cm lobulated, circumscribed mass was shown on her recent mammogram, suggestive of a fibroadenoma, with no evidence of malignancy (Figure 1). Ultrasound scanning showed a solid, hypoechoic and well-circumscribed mass, measuring 3.5 cm in diameter. However, elevated Ca 15-3 levels were found in blood tests she already had.

The same lesion, less than half in size, was originally shown on the first mammogram she had at her hometown four years before. It was not clinically palpable that time and it was thought to be a long existing benign fibroadenoma and was left in place.

She had full staging investigations (liver function tests, chest x-ray, computerized tomography scans of the chest and abdomen and bone scanning) which were all negative and the patient underwent an excisional biopsy, which revealed an invasive carcinoma. A left modified radical mastectomy followed.

### Pathological findings

On macroscopic examination the tumour measured 3.5 cm in diameter, had solid composition and polymorphic appearance, with tan and brown, pale and hemorrhagic areas.

**Figure 2:**
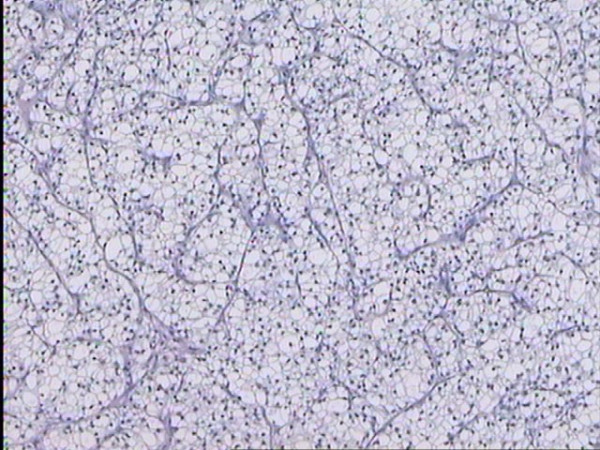
Clear-cell carcinoma of the breast, resembling clear-cell carcinoma of the kidney. The lobular arrangement and the fine vascular network are clearly visible.

Microscopic examination showed an invasive adenocarcinoma of the breast, characterized by average-sized cells, with well-defined borders and polygonal, rather than rounded contours. The neoplastic cells formed a matrix of solid, lobular, acinar and rarely papillary areas, with a fine vascular network in between (Figure 2). Foci of linear, trabecular and tubular growth patterns were visible. A few ducts with an intraductal (*in situ*) carcinoma of solid type were also noticed. The cytoplasm was clear and eccentrically placed and hyperchromatic nuclei with a low mitotic number (2 mitoses per 10HPF) were detected. Cells with mildly eosinophilic cytoplasma, nuclear pleomorphism and higher mitotic number were also seen. There was absence of necrosis and no lymphovascular invasion was noticed.

On histochemical examination, many of the above cells were positive for PAS staining erased by diastase pre-treatment, keratins AE1/AE3, EMA and Cytokeratin 7, but negative for c-erb-b2 (score 0), Cytokeratin 20, melanosomes and vimentin. Markers of myoepithelial cells were also negative: smooth muscle actin-SMA and S-100 (only a few isolated positive cells). Staining for Chromogranin was positive in some cells and for Synaptophysin in most cells, indicating a degree of neuroendocrine activity of the tumor. The tumor was strongly positive for estrogen receptors (ER) and negative for progesterone receptors (PgR).

Mastectomy specimen showed no residual neoplastic cells, and all 14 axillary lymph nodes removed were histologically tumor-free.

Hormonal therapy with the aromatase inactivator exemestane was started postoperatively and the patient is disease-free 48 months now.

## Discussion

Glycogen-rich clear cell carcinoma of the breast is a rare tumor. It is, however, the most frequent cause of clear cell morphology in breast malignancies [5]. It is composed of cells containing abundant glycogen, which is extracted when the tissue is processed for histological sections, leaving vacuolated cytoplasm. Extraction of the cytoplasmic components also occurs in lipid-rich carcinoma, signet-ring cell carcinoma and in some secretory variants of ductal or lobular carcinomas, as well as sebaceous, myoepithelial and endocrine tumours [5]. Cells with clear, vacuolated cytoplasm have been rarely found in benign breast lesions, such as clear cell hindradenoma, eccrine spiradenoma, acrospiroma and benign mammary myoepithelioma [4]. These are only known as isolated case reports.

Signet-ring carcinomas frequently coexist with ductal or lobular carcinomas and display an aggressive course with frequent lymph node and distant metastases [6]. Lipid-rich carcinomas tend to occur in elderly women as pure lesions, often involving axillary lymph nodes and have been occasionally reported to metastasize to the eyelid [7]. Secretory carcinomas frequently arise in young women, but rarely metastasize to axillary lymph nodes [8].

Clear cell neoplasms arise throughout the body. The vacuolated cytoplasm in many of these tumors can be attributed to large quantities of glycogen, as in clear cell carcinomas of the vagina, cervix, endometrium, ovary and salivary glands. The clear cell in renal adenocarcinoma contains not only glycogen, but abundant fat, both of which contribute to their optically clear quality [9]. In the lung, two clear cell tumors are known: the benign clear cell (sugar) tumor, which contains abundant glycogen [10,11] and the clear carcinoma, which contains abundant mucin [12]. Clear cell carcinoma of the larynx, a variant of mucoepidermoid carcinoma, gains its clear cell features from both intracytoplasmic glycogen and mucin [13]. In the thyroid, some clear cell carcinomas contain abundant colloid material [14], while others contain abundant glycogen [15]. Thus, the subcellular determinants of the clear cytoplasm vary from case to case.

Fewer than fifty cases of glycogen-rich clear cell carcinoma of the breast have been described since the first case was reported in 1981 [3]. The patients, aging from 35 to 78 years, presented with a mass that was sometimes accompanied by skin dimpling, nipple retraction or pain. Most tumours reported measure between 2 and 5 cm in diameter, with the largest lesion found to be 10 cm on clinical examination [3]. Hormone receptor analysis revealed that about 50% of the tumors were estrogen receptor positive, but all lesions studied, including our patient, have been negative for progesterone receptor. When analysed by flow cytometry, the tumors have been nondiploid [2].

Almost all patients were treated with mastectomy and axillary dissection and more than half had metastatic tumour in the axillary lymph nodes [3]. Our patient found to have negative axilla, despite the large size of the primary tumour.

The prognosis of glycogen-rich clear cell carcinoma of the breast is reported to be not particularly favorable and may be similar to or worse than that of ordinary invasive ductal carcinoma, when compared on a stage-matched basis [5]. However, in the case reported here, the patient had a history of at least 4 years with a slow growing clear cell carcinoma of her breast. The tumour had benign features on mammography and the well circumscribed appearance was suggestive of fibroadenoma. No axillary lymph node involvement was found and there was no evidence of systemic disease in staging investigations. The only pathologic finding before surgical treatment was elevated levels of Ca 15-3, which dropped to normal following operation. Our patient, staged T2N0M0, is free of disease 48 months now and continues adjuvant therapy with an aromatase inhibitor only.

## Conclusion

Glycogen-rich clear cell carcinoma of the breast is a rare tumor, its clinical behavior reported to be rather aggressive so far, might varies depending on special characteristics such as low grade and strongly positive ER expression as in the case of our patient.

## Competing interests

The authors declare that they have no competing interests.

## Authors' contributions

CM: drafting and revision of the manuscript, DM: Helped in preparation and revision of the manuscript, TP: revision of the manuscript and preparation of histology and immunoassays, EK: Surgery and follow-up and helped in revision of the manuscript; ZA: surgery and follow-up of patient and helped in preparation of the manuscript, MLH: editing of the manuscript for its scientific content, EG: surgery of the patient and revision of the manuscript for its scientific content. All authors read and approved the final manuscript.
